# Poly[[bis­(2,2-bipyridine)­bis­[μ_6_-5-(carboxyl­atometh­oxy)benzene-1,3-dicarboxyl­ato]trimanganese(II)] monohydrate]

**DOI:** 10.1107/S1600536811002819

**Published:** 2011-01-26

**Authors:** Zhan-Guo Jiang, Jing Chen, Yun-Long Feng

**Affiliations:** a Zhejiang Key Laboratory for Reactive Chemistry on Solid Surfaces, College of Chemistry and Life Science, Zhejiang Normal University, Jinhua, Zhejiang 321004, People’s Republic of China

## Abstract

The title compound, {[Mn_3_(C_10_H_5_O_7_)_2_(C_10_H_8_N_2_)_2_]·H_2_O}_*n*_, was synthesized under hydro­thermal conditions. Six carboxyl­ate groups of six 5-(carboxyl­atometh­oxy)benzene-1,3-dicarboxyl­ate anions (OABDC^3−^) join three Mn^II^ ions into a trinuclear centrosymmetric [Mn_3_(μ_2_-COO)_6_] unit with one Mn site situated on a centre of inversion. The latter Mn^II^ ion exhibits a distorted MnO_6_ coordination, whereas the other Mn^II^ ion has a trigonal–bipyramidal MnN_2_O_3_ coordination environment resulting from three carboxylate O atoms and the two N atoms of the bipyridine ligand. Adjacent units are linked to each other by OABDC^3−^ ligands into a layer parallel to (010). Within the layer, O—H⋯O hydrogen-bonding inter­actions involving the uncoordinated and half-occupied water mol­ecule and the free carboxyl­ate O atoms are observed. The layers stack along [010], constructing a three-dimensional structure through π–π inter­actions between adjacent pyridine rings, with a centroid–centroid distance of 3.473 (5) Å.

## Related literature

For the construction of metal-organic frameworks with polycarboxyl­ate ligands, see: Xing *et al.* (2010 ▶); Cao *et al.* (2004 ▶, 2007 ▶).
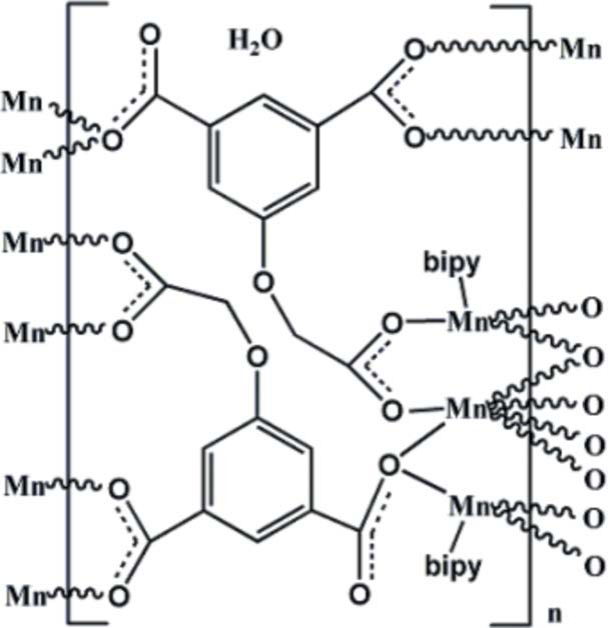


## Experimental

### 

#### Crystal data

[Mn_3_(C_10_H_5_O_7_)_2_(C_10_H_8_N_2_)_2_]·H_2_O
*M* *_r_* = 969.48
Monoclinic, 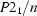

*a* = 8.5683 (1) Å
*b* = 25.2280 (4) Å
*c* = 9.7685 (1) Å
β = 114.633 (1)°
*V* = 1919.41 (4) Å^3^
*Z* = 2
Mo *K*α radiation
μ = 1.05 mm^−1^
*T* = 296 K
0.37 × 0.19 × 0.09 mm

#### Data collection

Bruker APEXII area-detector diffractometer
Absorption correction: multi-scan (*SADABS*; Sheldrick, 1996 ▶) *T*_min_ = 0.786, *T*_max_ = 0.907
16528 measured reflections
3350 independent reflections
3012 reflections with *I* > 2σ(*I*)
*R*_int_ = 0.023

#### Refinement

*R*[*F* ^2^ > 2σ(*F* ^2^)] = 0.036
*wR*(*F* ^2^) = 0.123
*S* = 1.10
3350 reflections
292 parameters
6 restraints
H atoms treated by a mixture of independent and constrained refinement
Δρ_max_ = 1.11 e Å^−3^
Δρ_min_ = −0.47 e Å^−3^

### 

Data collection: *APEX2* (Bruker, 2006 ▶); cell refinement: *SAINT* (Bruker, 2006 ▶); data reduction: *SAINT*; program(s) used to solve structure: *SHELXS97* (Sheldrick, 2008 ▶); program(s) used to refine structure: *SHELXL97* (Sheldrick, 2008 ▶); molecular graphics: *DIAMOND* (Brandenburg, 2008 ▶); software used to prepare material for publication: *SHELXTL* (Sheldrick, 2008 ▶).

## Supplementary Material

Crystal structure: contains datablocks I, global. DOI: 10.1107/S1600536811002819/wm2447sup1.cif

Structure factors: contains datablocks I. DOI: 10.1107/S1600536811002819/wm2447Isup2.hkl

Additional supplementary materials:  crystallographic information; 3D view; checkCIF report

## Figures and Tables

**Table 1:** Selected bond lengths (Å)

Mn1—O7^i^	2.100 (2)
Mn1—O3^ii^	2.148 (2)
Mn1—O1	2.175 (2)
Mn1—N2	2.228 (3)
Mn1—N1	2.251 (3)
Mn2—O4^iii^	2.108 (2)
Mn2—O6^iv^	2.159 (2)
Mn2—O1	2.322 (2)

Symmetry codes: (i) 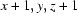; (ii) 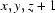; (iii) 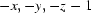; (iv) 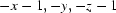.

**Table 2:** Hydrogen-bond geometry (Å, °)

*D*—H⋯*A*	*D*—H	H⋯*A*	*D*⋯*A*	*D*—H⋯*A*
O1*W*—H1*WB*⋯O2^iii^	0.85 (1)	2.19 (2)	3.015 (5)	164 (6)

Symmetry code: (iii) 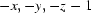.

**Fig. 1.**
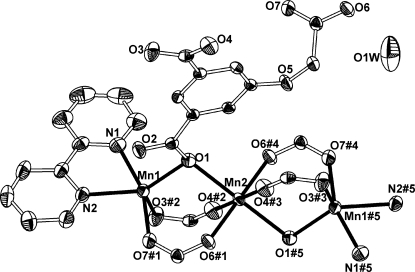
The molecular structure of title the compound. Displacement ellipsoids are drawn at the 30% probability level. [Symmetry codes: (1) x + 1,y,z + 1; (2) x,y,z + 1; (3) -x,-y,-z - 1; (4) -x - 1,-y,-z - 1; (5) -x,-y,-z.]

**Fig. 2.**
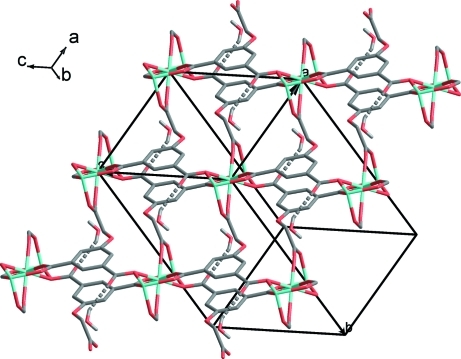
two-dimensional layered structure (bipys are omitted for clarity).

**Fig. 3.**
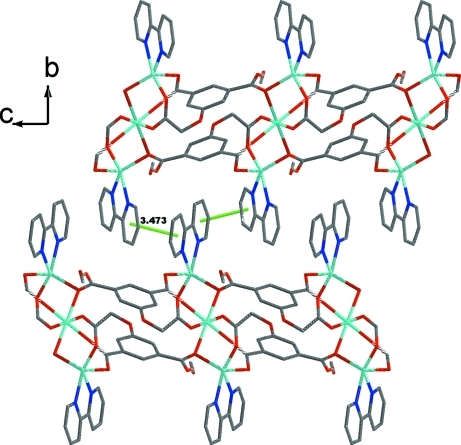
View of π–π interactions between layers.

## supplementary crystallographic information

### Comment

Aromatic polycarboxylic acids like 1,3,5-benzenetricarboxylate (H_3_btc) and 1,2,4,5-benzenetetracarboxylate (H_4_btec) are widely used to construct metal-organic frameworks (Xing *et al.*, 2010). Aromatic polycarboxylate ligands with rigid and flexible carboxyl group are less reported. 5-oxyacetato-1,3-benzenebiscarboxylic acid (H_3_OABDC) is a ligand with two rigid carboxyl groups and one flexible oxyacetato group. Previous structural studies including this ligand have also been reported (Cao *et al.*, 2004, 2007).

The title compound, [Mn_3_(C_10_H_5_O_7_)_2_(C_10_H_8_N_2_)_2_]^.^H_2_O, crystallizes with two Mn^II^ ions (Mn1 on a general position, Mn2 on a centre of inversion), one OABDC^3-^ trianion, one chelating 2,2-bipyridine (bipy) ligand and one water molecule (half-occupied) in the asymmetric unit. Mn1 is five-coordinated by two N atoms from the bipy ligand, one O atom from one flexible carboxyl group of the OABDC^3-^ anion and two O atoms from two rigid carboxyl groups of the OABDC^3-^ anion to form a distorted trigonal-bipyramidal environment. Mn2 is six-coordinated by two O atoms from two flexible carboxyl groups of the OABDC^3-^ anion and four oxygen atoms from four rigid carboxyl groups of adjacent OABDC^3-^ anions to form a distorted octahedral environment (Table 1, Fig. 1).

Six OABDC^3-^ anions join three Mn^II^ ions through their carboxyl groups. As a result, trinuclear [Mn_3_(µ_2_-COO)_6_]_n_ layers are formed in which Mn2 sits on a centre of inversion of each trinuclear unit. These layers are oriented parallel to (010). Uncoordinated water molecules interact with non-bonded O atoms of the carboxylate groups within the layers through relatively weak O—H···O hydrogen-bonding interactions (Table 2, Fig. 2).

The bipy ligands decorate the top and bottom of each layer. Between parallel pyridine rings of adjacent layers exist π–π interactions with a centroid- centroid distance of 3.473 (5) Å that consolidate the packing of the structure along [010] into a three-dimensional framework (Fig. 3).

### Experimental

All reagents were purchased commercially and used without further purification. A mixture of 5-oxyacetato-1,3-benzenebiscarboxylic acid (H_3_OABDC; 0.1205 g, 0.5 mmol), MnCl_2_^.^4H_2_O (0.0976 g, 0.5 mmol), bipyridine (0.0786 g, 0.5 mmol), was dissolved with NaOH (0.0101 g, 0.25 mmol) in water (15 ml) and loaded in a 25 ml stainless steel reactor with a telflon liner. The autoclave was heated at 433 K for 72 h, and then cooled to room temperature over 3 days with a cooling rate of 5 K per hour. Yellow single crystals of the title compound were obtained by slow evaporation of the filtrate over a few days.

### Refinement

The carbon-bound H-atoms were positioned geometrically and included in the refinement using a riding model [C—H 0.93 for aromatic C atoms and 0.97Å for methylen C atoms with *U*_iso_(H) = 1.2*U*_eq_(C)]. The water H-atoms were located in a difference Fourier map and refined with an O—H distance restrained to 0.85 (2)Å [*U*_iso_(H) = 1.2*U*_eq_(O)]. The lattice water (O1W) molecule shows half-occupation.

### Crystal data

**Table:**

[Mn_3_(C_10_H_5_O_7_)_2_(C_10_H_8_N_2_)_2_]·H_2_O	*F*(000) = 982
*M**_r_* = 969.48	*D*_x_ = 1.677 Mg m^−^^3^
Monoclinic, *P*2_1_/*n*	Mo *K*α radiation, λ = 0.71073 Å
Hall symbol: -P 2yn	Cell parameters from 8045 reflections
*a* = 8.5683 (1) Å	θ = 2.4–27.7°
*b* = 25.2280 (4) Å	µ = 1.05 mm^−^^1^
*c* = 9.7685 (1) Å	*T* = 296 K
β = 114.633 (1)°	Block, yellow
*V* = 1919.41 (4) Å^3^	0.37 × 0.19 × 0.09 mm
*Z* = 2	

### Data collection

**Table:**

Bruker APEXII area-detector diffractometer	3350 independent reflections
Radiation source: fine-focus sealed tube	3012 reflections with *I* > 2σ(*I*)
graphite	*R*_int_ = 0.023
ω scans	θ_max_ = 25.0°, θ_min_ = 2.4°
Absorption correction: multi-scan (*SADABS*; Sheldrick, 1996)	*h* = −9→8
*T*_min_ = 0.786, *T*_max_ = 0.907	*k* = −24→30
16528 measured reflections	*l* = −11→11

### Refinement

**Table:**

Refinement on *F*^2^	Primary atom site location: structure-invariant direct methods
Least-squares matrix: full	Secondary atom site location: difference Fourier map
*R*[*F*^2^ > 2σ(*F*^2^)] = 0.036	Hydrogen site location: inferred from neighbouring sites
*wR*(*F*^2^) = 0.123	H atoms treated by a mixture of independent and constrained refinement
*S* = 1.10	*w* = 1/[σ^2^(*F*_o_^2^) + (0.0743*P*)^2^ + 1.9128*P*] where *P* = (*F*_o_^2^ + 2*F*_c_^2^)/3
3350 reflections	(Δ/σ)_max_ < 0.001
292 parameters	Δρ_max_ = 1.11 e Å^−^^3^
6 restraints	Δρ_min_ = −0.47 e Å^−^^3^

### Special details

**Table:**

Geometry. All e.s.d.'s (except the e.s.d. in the dihedral angle between two l.s. planes) are estimated using the full covariance matrix. The cell e.s.d.'s are taken into account individually in the estimation of e.s.d.'s in distances, angles and torsion angles; correlations between e.s.d.'s in cell parameters are only used when they are defined by crystal symmetry. An approximate (isotropic) treatment of cell e.s.d.'s is used for estimating e.s.d.'s involving l.s. planes.
Refinement. Refinement of *F*^2^ against ALL reflections. The weighted *R*-factor *wR* and goodness of fit *S* are based on *F*^2^, conventional *R*-factors *R* are based on *F*, with *F* set to zero for negative *F*^2^. The threshold expression of *F*^2^ > σ(*F*^2^) is used only for calculating *R*-factors(gt) *etc*. and is not relevant to the choice of reflections for refinement. *R*-factors based on *F*^2^ are statistically about twice as large as those based on *F*, and *R*- factors based on ALL data will be even larger.

### Fractional atomic coordinates and isotropic or equivalent isotropic displacement parameters (Å^2^)

**Table:**

	*x*	*y*	*z*	*U*_iso_*/*U*_eq_	Occ. (<1)
Mn1	0.23443 (6)	0.121021 (17)	0.11506 (5)	0.02566 (17)	
Mn2	0.0000	0.0000	0.0000	0.02189 (18)	
O1W	−0.6212 (6)	−0.12729 (17)	−0.9008 (6)	0.0426 (12)	0.50
H1WB	−0.5137 (17)	−0.130 (3)	−0.874 (7)	0.051*	0.50
H1WA	−0.659 (7)	−0.111 (3)	−0.984 (5)	0.051*	0.50
O1	0.0588 (3)	0.08005 (8)	−0.0861 (2)	0.0297 (5)	
O2	0.2531 (3)	0.12144 (9)	−0.1396 (2)	0.0353 (5)	
O3	0.1568 (3)	0.11891 (9)	−0.7023 (3)	0.0416 (6)	
O4	0.0038 (3)	0.04991 (9)	−0.8255 (2)	0.0387 (6)	
O5	−0.3626 (3)	0.00513 (9)	−0.5459 (2)	0.0337 (5)	
O6	−0.7274 (3)	−0.01469 (9)	−0.8866 (2)	0.0337 (5)	
O7	−0.5860 (3)	0.06004 (9)	−0.7894 (3)	0.0408 (6)	
N1	0.1153 (4)	0.20221 (11)	0.0619 (3)	0.0344 (6)	
N2	0.4486 (3)	0.17919 (10)	0.1761 (3)	0.0308 (6)	
C1	0.0167 (3)	0.08164 (10)	−0.3438 (3)	0.0207 (6)	
C2	0.0778 (4)	0.09280 (10)	−0.4528 (3)	0.0213 (6)	
H2	0.1769	0.1128	−0.4285	0.026*	
C3	−0.0115 (4)	0.07361 (10)	−0.5978 (3)	0.0217 (6)	
C4	−0.1610 (4)	0.04408 (11)	−0.6359 (3)	0.0227 (6)	
H4	−0.2187	0.0308	−0.7330	0.027*	
C5	−0.2231 (3)	0.03463 (11)	−0.5281 (3)	0.0228 (6)	
C6	−0.1349 (4)	0.05419 (11)	−0.3831 (3)	0.0232 (6)	
H6	−0.1786	0.0487	−0.3116	0.028*	
C7	0.1182 (4)	0.09562 (10)	−0.1816 (3)	0.0237 (6)	
C8	0.0547 (4)	0.08148 (12)	−0.7170 (3)	0.0269 (6)	
C9	−0.4589 (4)	−0.02186 (12)	−0.6826 (3)	0.0306 (7)	
H9A	−0.5073	−0.0537	−0.6605	0.037*	
H9B	−0.3821	−0.0326	−0.7277	0.037*	
C10	−0.6025 (4)	0.01101 (12)	−0.7951 (3)	0.0264 (6)	
C11	−0.0531 (5)	0.21147 (17)	0.0082 (4)	0.0468 (9)	
H11	−0.1278	0.1827	−0.0174	0.056*	
C12	−0.1217 (6)	0.2624 (2)	−0.0112 (4)	0.0587 (12)	
H12	−0.2397	0.2678	−0.0514	0.070*	
C13	−0.0103 (6)	0.30430 (18)	0.0307 (5)	0.0613 (12)	
H13	−0.0521	0.3388	0.0210	0.074*	
C14	0.1621 (6)	0.29541 (15)	0.0868 (4)	0.0514 (10)	
H14	0.2382	0.3238	0.1155	0.062*	
C15	0.2239 (4)	0.24397 (13)	0.1008 (3)	0.0342 (7)	
C16	0.4077 (4)	0.23094 (12)	0.1557 (3)	0.0318 (7)	
C17	0.5338 (5)	0.26898 (13)	0.1875 (4)	0.0440 (9)	
H17	0.5040	0.3046	0.1724	0.053*	
C18	0.7043 (5)	0.25413 (15)	0.2416 (4)	0.0479 (9)	
H18	0.7899	0.2796	0.2644	0.057*	
C19	0.7455 (5)	0.20131 (15)	0.2613 (4)	0.0456 (9)	
H19	0.8589	0.1900	0.2968	0.055*	
C20	0.6131 (4)	0.16548 (13)	0.2268 (4)	0.0381 (8)	
H20	0.6403	0.1296	0.2397	0.046*	

### Atomic displacement parameters (Å^2^)

**Table:**

	*U*^11^	*U*^22^	*U*^33^	*U*^12^	*U*^13^	*U*^23^
Mn1	0.0318 (3)	0.0250 (3)	0.0198 (3)	−0.00594 (17)	0.0103 (2)	−0.00228 (16)
Mn2	0.0203 (3)	0.0264 (3)	0.0174 (3)	−0.0036 (2)	0.0062 (2)	−0.0038 (2)
O1W	0.031 (2)	0.022 (2)	0.081 (4)	−0.0176 (19)	0.030 (3)	−0.026 (2)
O1	0.0381 (12)	0.0346 (11)	0.0175 (9)	−0.0066 (9)	0.0128 (9)	−0.0019 (8)
O2	0.0355 (13)	0.0405 (13)	0.0266 (11)	−0.0142 (10)	0.0096 (10)	−0.0066 (9)
O3	0.0529 (15)	0.0437 (14)	0.0397 (13)	−0.0147 (11)	0.0308 (12)	−0.0004 (10)
O4	0.0457 (14)	0.0523 (14)	0.0230 (11)	−0.0026 (11)	0.0191 (10)	−0.0108 (10)
O5	0.0225 (11)	0.0500 (13)	0.0265 (11)	−0.0143 (9)	0.0081 (9)	−0.0015 (9)
O6	0.0240 (11)	0.0355 (12)	0.0331 (11)	−0.0025 (9)	0.0036 (9)	−0.0045 (9)
O7	0.0329 (12)	0.0268 (12)	0.0507 (14)	−0.0042 (9)	0.0056 (11)	0.0036 (10)
N1	0.0399 (16)	0.0386 (15)	0.0291 (13)	0.0006 (12)	0.0188 (12)	0.0027 (11)
N2	0.0389 (15)	0.0249 (13)	0.0306 (13)	−0.0035 (11)	0.0164 (12)	−0.0029 (10)
C1	0.0251 (14)	0.0191 (13)	0.0180 (13)	0.0014 (11)	0.0091 (11)	0.0008 (10)
C2	0.0230 (14)	0.0194 (13)	0.0215 (13)	−0.0018 (11)	0.0093 (11)	−0.0003 (10)
C3	0.0256 (14)	0.0219 (14)	0.0192 (13)	0.0039 (11)	0.0110 (11)	0.0018 (10)
C4	0.0222 (14)	0.0259 (14)	0.0168 (12)	−0.0006 (11)	0.0050 (11)	−0.0017 (10)
C5	0.0181 (13)	0.0262 (14)	0.0225 (13)	0.0007 (11)	0.0069 (11)	0.0039 (11)
C6	0.0233 (14)	0.0288 (14)	0.0181 (13)	0.0016 (11)	0.0091 (11)	0.0034 (11)
C7	0.0302 (15)	0.0199 (13)	0.0200 (13)	0.0019 (12)	0.0096 (12)	−0.0011 (11)
C8	0.0298 (16)	0.0321 (16)	0.0214 (14)	0.0043 (13)	0.0132 (12)	0.0056 (12)
C9	0.0236 (15)	0.0301 (16)	0.0330 (16)	−0.0061 (12)	0.0067 (13)	−0.0023 (13)
C10	0.0220 (15)	0.0307 (16)	0.0273 (15)	−0.0029 (12)	0.0111 (12)	−0.0002 (12)
C11	0.044 (2)	0.062 (2)	0.0393 (19)	0.0037 (18)	0.0220 (16)	0.0068 (17)
C12	0.057 (2)	0.085 (3)	0.042 (2)	0.029 (2)	0.0267 (19)	0.013 (2)
C13	0.081 (3)	0.052 (3)	0.053 (2)	0.029 (2)	0.030 (2)	0.008 (2)
C14	0.068 (3)	0.0360 (19)	0.051 (2)	0.0100 (19)	0.025 (2)	0.0001 (16)
C15	0.050 (2)	0.0301 (16)	0.0258 (15)	0.0028 (14)	0.0192 (14)	0.0017 (12)
C16	0.0471 (19)	0.0265 (15)	0.0246 (15)	−0.0049 (14)	0.0177 (14)	−0.0018 (12)
C17	0.063 (3)	0.0251 (16)	0.048 (2)	−0.0093 (16)	0.0268 (18)	−0.0055 (14)
C18	0.053 (2)	0.046 (2)	0.052 (2)	−0.0217 (18)	0.0289 (18)	−0.0113 (17)
C19	0.041 (2)	0.048 (2)	0.051 (2)	−0.0097 (17)	0.0229 (17)	−0.0070 (17)
C20	0.0378 (19)	0.0321 (17)	0.0450 (19)	−0.0029 (14)	0.0179 (15)	−0.0035 (14)

### Geometric parameters (Å, °)

**Table:**

Mn1—O7^i^	2.100 (2)	C1—C2	1.396 (4)
Mn1—O3^ii^	2.148 (2)	C1—C7	1.498 (4)
Mn1—O1	2.175 (2)	C2—C3	1.386 (4)
Mn1—N2	2.228 (3)	C2—H2	0.9300
Mn1—N1	2.251 (3)	C3—C4	1.392 (4)
Mn2—O4^ii^	2.108 (2)	C3—C8	1.506 (4)
Mn2—O4^iii^	2.108 (2)	C4—C5	1.384 (4)
Mn2—O6^iv^	2.159 (2)	C4—H4	0.9300
Mn2—O6^i^	2.159 (2)	C5—C6	1.389 (4)
Mn2—O1^v^	2.322 (2)	C6—H6	0.9300
Mn2—O1	2.322 (2)	C9—C10	1.511 (4)
O1W—H1WB	0.849 (2)	C9—H9A	0.9700
O1W—H1WA	0.849 (2)	C9—H9B	0.9700
O1—C7	1.296 (3)	C11—C12	1.394 (6)
O2—C7	1.238 (4)	C11—H11	0.9300
O3—C8	1.254 (4)	C12—C13	1.366 (7)
O3—Mn1^vi^	2.148 (2)	C12—H12	0.9300
O4—C8	1.250 (4)	C13—C14	1.363 (6)
O4—Mn2^vi^	2.108 (2)	C13—H13	0.9300
O5—C5	1.356 (3)	C14—C15	1.387 (5)
O5—C9	1.417 (4)	C14—H14	0.9300
O6—C10	1.252 (4)	C15—C16	1.474 (5)
O6—Mn2^vii^	2.159 (2)	C16—C17	1.380 (5)
O7—C10	1.244 (4)	C17—C18	1.382 (6)
O7—Mn1^vii^	2.100 (2)	C17—H17	0.9300
N1—C11	1.334 (5)	C18—C19	1.371 (5)
N1—C15	1.351 (4)	C18—H18	0.9300
N2—C20	1.330 (4)	C19—C20	1.378 (5)
N2—C16	1.345 (4)	C19—H19	0.9300
C1—C6	1.378 (4)	C20—H20	0.9300
			
O7^i^—Mn1—O3^ii^	92.04 (10)	O5—C5—C4	126.5 (2)
O7^i^—Mn1—O1	99.00 (9)	O5—C5—C6	113.7 (2)
O3^ii^—Mn1—O1	113.65 (9)	C4—C5—C6	119.7 (3)
O7^i^—Mn1—N2	89.68 (9)	C1—C6—C5	120.8 (3)
O3^ii^—Mn1—N2	107.44 (9)	C1—C6—H6	119.6
O1—Mn1—N2	137.52 (8)	C5—C6—H6	119.6
O7^i^—Mn1—N1	161.31 (10)	O2—C7—O1	120.9 (2)
O3^ii^—Mn1—N1	86.93 (9)	O2—C7—C1	121.6 (2)
O1—Mn1—N1	98.49 (9)	O1—C7—C1	117.5 (2)
N2—Mn1—N1	72.92 (10)	O4—C8—O3	123.8 (3)
O4^ii^—Mn2—O4^iii^	180.00 (12)	O4—C8—C3	117.5 (3)
O4^ii^—Mn2—O6^iv^	87.76 (9)	O3—C8—C3	118.7 (3)
O4^iii^—Mn2—O6^iv^	92.24 (9)	O5—C9—C10	113.6 (2)
O4^ii^—Mn2—O6^i^	92.24 (9)	O5—C9—H9A	108.9
O4^iii^—Mn2—O6^i^	87.76 (9)	C10—C9—H9A	108.9
O6^iv^—Mn2—O6^i^	180.00 (11)	O5—C9—H9B	108.9
O4^ii^—Mn2—O1^v^	99.16 (8)	C10—C9—H9B	108.9
O4^iii^—Mn2—O1^v^	80.84 (8)	H9A—C9—H9B	107.7
O6^iv^—Mn2—O1^v^	89.06 (8)	O7—C10—O6	126.5 (3)
O6^i^—Mn2—O1^v^	90.94 (8)	O7—C10—C9	118.0 (3)
O4^ii^—Mn2—O1	80.84 (8)	O6—C10—C9	115.5 (3)
O4^iii^—Mn2—O1	99.16 (8)	N1—C11—C12	122.7 (4)
O6^iv^—Mn2—O1	90.94 (8)	N1—C11—H11	118.6
O6^i^—Mn2—O1	89.06 (8)	C12—C11—H11	118.6
O1^v^—Mn2—O1	180.00 (5)	C13—C12—C11	118.1 (4)
H1WB—O1W—H1WA	105.3 (4)	C13—C12—H12	121.0
C7—O1—Mn1	100.06 (17)	C11—C12—H12	121.0
C7—O1—Mn2	137.20 (18)	C14—C13—C12	119.8 (4)
Mn1—O1—Mn2	105.02 (8)	C14—C13—H13	120.1
C8—O3—Mn1^vi^	111.51 (19)	C12—C13—H13	120.1
C8—O4—Mn2^vi^	161.5 (2)	C13—C14—C15	119.9 (4)
C5—O5—C9	121.3 (2)	C13—C14—H14	120.0
C10—O6—Mn2^vii^	134.2 (2)	C15—C14—H14	120.0
C10—O7—Mn1^vii^	131.3 (2)	N1—C15—C14	120.8 (3)
C11—N1—C15	118.6 (3)	N1—C15—C16	115.8 (3)
C11—N1—Mn1	124.2 (3)	C14—C15—C16	123.4 (3)
C15—N1—Mn1	116.8 (2)	N2—C16—C17	120.7 (3)
C20—N2—C16	118.6 (3)	N2—C16—C15	116.3 (3)
C20—N2—Mn1	123.7 (2)	C17—C16—C15	123.0 (3)
C16—N2—Mn1	117.7 (2)	C16—C17—C18	120.1 (3)
C6—C1—C2	119.9 (2)	C16—C17—H17	120.0
C6—C1—C7	118.5 (2)	C18—C17—H17	120.0
C2—C1—C7	121.5 (2)	C19—C18—C17	119.1 (3)
C3—C2—C1	119.1 (2)	C19—C18—H18	120.5
C3—C2—H2	120.5	C17—C18—H18	120.5
C1—C2—H2	120.5	C18—C19—C20	117.8 (4)
C2—C3—C4	121.0 (2)	C18—C19—H19	121.1
C2—C3—C8	121.3 (2)	C20—C19—H19	121.1
C4—C3—C8	117.7 (2)	N2—C20—C19	123.8 (3)
C5—C4—C3	119.4 (2)	N2—C20—H20	118.1
C5—C4—H4	120.3	C19—C20—H20	118.1
C3—C4—H4	120.3		
			
O7^i^—Mn1—O1—C7	−94.71 (18)	Mn1—O1—C7—C1	−172.1 (2)
O3^ii^—Mn1—O1—C7	169.05 (17)	Mn2—O1—C7—C1	62.5 (4)
N2—Mn1—O1—C7	4.8 (2)	C6—C1—C7—O2	−178.1 (3)
N1—Mn1—O1—C7	78.69 (18)	C2—C1—C7—O2	6.0 (4)
O7^i^—Mn1—O1—Mn2	50.28 (10)	C6—C1—C7—O1	1.3 (4)
O3^ii^—Mn1—O1—Mn2	−45.96 (12)	C2—C1—C7—O1	−174.6 (2)
N2—Mn1—O1—Mn2	149.75 (11)	Mn2^vi^—O4—C8—O3	36.8 (8)
N1—Mn1—O1—Mn2	−136.32 (9)	Mn2^vi^—O4—C8—C3	−143.2 (6)
O4^ii^—Mn2—O1—C7	162.4 (3)	Mn1^vi^—O3—C8—O4	−0.7 (4)
O4^iii^—Mn2—O1—C7	−17.6 (3)	Mn1^vi^—O3—C8—C3	179.27 (19)
O6^iv^—Mn2—O1—C7	−110.0 (3)	C2—C3—C8—O4	157.0 (3)
O6^i^—Mn2—O1—C7	70.0 (3)	C4—C3—C8—O4	−19.8 (4)
O4^ii^—Mn2—O1—Mn1	38.66 (9)	C2—C3—C8—O3	−23.0 (4)
O4^iii^—Mn2—O1—Mn1	−141.34 (9)	C4—C3—C8—O3	160.2 (3)
O6^iv^—Mn2—O1—Mn1	126.24 (9)	C5—O5—C9—C10	−89.4 (3)
O6^i^—Mn2—O1—Mn1	−53.76 (9)	Mn1^vii^—O7—C10—O6	15.9 (5)
O7^i^—Mn1—N1—C11	−156.2 (3)	Mn1^vii^—O7—C10—C9	−164.6 (2)
O3^ii^—Mn1—N1—C11	−68.9 (3)	Mn2^vii^—O6—C10—O7	−32.8 (5)
O1—Mn1—N1—C11	44.5 (3)	Mn2^vii^—O6—C10—C9	147.6 (2)
N2—Mn1—N1—C11	−178.2 (3)	O5—C9—C10—O7	27.3 (4)
O7^i^—Mn1—N1—C15	17.3 (4)	O5—C9—C10—O6	−153.1 (3)
O3^ii^—Mn1—N1—C15	104.6 (2)	C15—N1—C11—C12	0.8 (5)
O1—Mn1—N1—C15	−141.9 (2)	Mn1—N1—C11—C12	174.2 (3)
N2—Mn1—N1—C15	−4.7 (2)	N1—C11—C12—C13	−1.7 (6)
O7^i^—Mn1—N2—C20	9.9 (3)	C11—C12—C13—C14	1.2 (6)
O3^ii^—Mn1—N2—C20	102.0 (3)	C12—C13—C14—C15	0.1 (6)
O1—Mn1—N2—C20	−93.1 (3)	C11—N1—C15—C14	0.6 (4)
N1—Mn1—N2—C20	−177.0 (3)	Mn1—N1—C15—C14	−173.3 (2)
O7^i^—Mn1—N2—C16	−172.2 (2)	C11—N1—C15—C16	−178.5 (3)
O3^ii^—Mn1—N2—C16	−80.2 (2)	Mn1—N1—C15—C16	7.6 (3)
O1—Mn1—N2—C16	84.7 (2)	C13—C14—C15—N1	−1.1 (5)
N1—Mn1—N2—C16	0.9 (2)	C13—C14—C15—C16	178.0 (3)
C6—C1—C2—C3	−3.2 (4)	C20—N2—C16—C17	−0.4 (4)
C7—C1—C2—C3	172.6 (2)	Mn1—N2—C16—C17	−178.3 (2)
C1—C2—C3—C4	0.7 (4)	C20—N2—C16—C15	−179.4 (3)
C1—C2—C3—C8	−176.0 (2)	Mn1—N2—C16—C15	2.7 (3)
C2—C3—C4—C5	1.3 (4)	N1—C15—C16—N2	−6.8 (4)
C8—C3—C4—C5	178.1 (2)	C14—C15—C16—N2	174.1 (3)
C9—O5—C5—C4	2.6 (4)	N1—C15—C16—C17	174.2 (3)
C9—O5—C5—C6	−174.3 (2)	C14—C15—C16—C17	−4.9 (5)
C3—C4—C5—O5	−177.4 (3)	N2—C16—C17—C18	−0.3 (5)
C3—C4—C5—C6	−0.7 (4)	C15—C16—C17—C18	178.6 (3)
C2—C1—C6—C5	3.8 (4)	C16—C17—C18—C19	0.8 (5)
C7—C1—C6—C5	−172.1 (2)	C17—C18—C19—C20	−0.5 (5)
O5—C5—C6—C1	175.2 (2)	C16—N2—C20—C19	0.6 (5)
C4—C5—C6—C1	−1.9 (4)	Mn1—N2—C20—C19	178.4 (3)
Mn1—O1—C7—O2	7.3 (3)	C18—C19—C20—N2	−0.2 (6)
Mn2—O1—C7—O2	−118.1 (3)		

Symmetry codes: (i) *x*+1, *y*, *z*+1; (ii) *x*, *y*, *z*+1; (iii) −*x*, −*y*, −*z*−1; (iv) −*x*−1, −*y*, −*z*−1; (v) −*x*, −*y*, −*z*; (vi) *x*, *y*, *z*−1; (vii) *x*−1, *y*, *z*−1.

### Hydrogen-bond geometry (Å, °)

**Table:**

*D*—H···*A*	*D*—H	H···*A*	*D*···*A*	*D*—H···*A*
O1W—H1WB···O2^iii^	0.85 (1)	2.19 (2)	3.015 (5)	164 (6)

Symmetry codes: (iii) −*x*, −*y*, −*z*−1.
